# Does Screening for Bipolar Disorders Identify a “Dysregulation of Mood, Energy, and Social Rhythms Syndrome” (DYMERS)? A Heuristic Working Hypothesis

**DOI:** 10.3390/jcm12155162

**Published:** 2023-08-07

**Authors:** Mauro Giovanni Carta, Goce Kalcev, Michele Fornaro, Samantha Pinna, Cesar Ivan Aviles Gonzalez, Antonio Egidio Nardi, Diego Primavera

**Affiliations:** 1 Department of Medical Sciences and Public Health, University of Cagliari, Monserrato Blocco I (CA), 09042 Cagliari, Italy; maurogcarta@gmail.com (M.G.C.); samanthapinna1984@gmail.com (S.P.); diego.primavera@tiscali.it (D.P.); 2 Department of Psychiatry, Federico II University of Naples, 80126 Naples, Italy; dott.fornaro@gmail.com; 3 Nursing Program, Faculty of Health Sciences, Universidad Popular del Cesar, Sede Sabanas, Valledupar 20002, Colombia; infermiere2020@gmail.com; 4 Laboratory Panic and Respiration, Institute of Psychiatry (IPUB), Federal University of Rio de Janeiro (UFRJ), Rio de Janeiro 22725, Brazil; antonioenardi@gmail.com

**Keywords:** DYMERS, SF-12, MDQ, social rhythms, mood disorders, comorbidities

## Abstract

The aim of this paper is to verify if people with a positive score on the Mood Disorder Questionnaire (MDQ) without comorbidity of mood disorders showed a worse level of Health-related Quality of life (HRQol) compared to a control-matched sample of MDQ negatives, identifying a specific syndrome. This is a case-control study based on a database from a community survey. Cases: MDQ-positive without mood disorders; Controls: MDQ negatives matched by sex, age, and psychiatric diagnosis according to the Diagnostic and Statistical Manual of Mental Disorders IV (DSM-IV) criteria. Tools: MDQ, the Advanced Neuropsychiatric Tools and Assessment Schedule (ANTAS) semi-structured interview for psychiatric diagnosis, and the Health Survey Short Form (SF-12) for measuring HRQol. People scoring positive on the MDQ without a diagnosis of mood disorders showed significantly lower scores on the SF-12 compared to people of the same age and of the same sex with an equal diagnosis of psychiatric disorders not related to mood disorders (35.21 ± 6.30 vs. 41.48 ± 3.39, *p* < 0.0001). In the debate whether a positive score on the MDQ selects an area of “malaise” due to the presence of disorders differing from Bipolar Disorders, or if a positive score on the MDQ may be considered a “subthreshold” form of bipolar disorder in people who may later develop bipolar disorder, a third hypothesis can be advanced, i.e., that a positive score on the MDQ identifies a specific “Dysregulation of Mood, Energy, and Social Rhythms Syndrome” (DYMERS), characterized by a considerable amount of suffering and not attributable to other disorders, and which might represent a trigger for the previously mentioned disorders with which a positive score on the MDQ is associated, probably including, in severe conditions, bipolar disorder.

## 1. Introduction

The interest in validated rating tools for the detection of hypomania among people endorsing a major depressive episode saw a surge some decades ago [1]. The Hypomania Checklist (HCL-32) represents a core tool primarily adopted in clinical settings [2,3,4], whereas the Mood Disorder Questionnaire (MDQ) [5,6,7,8] is particularly, but not only, employed in epidemiological studies: surveys carried out in the community found a (lifetime) prevalence of around 4% in the US [6], of 3% in Italy [9], of 3.6% in France [10], of 18.6% in a large sample of college students [11], and of 4.3% in overall adults in South Korea [12]. In contrast, the prevalence rate recorded in community surveys using structured interviews was approximately under 2% [4]. MDQ has been used to demonstrate, among other things, a greater prevalence of bipolar spectrum features (also subthreshold) in people without a diagnosis of bipolar disorders with Major Depressive Disorder [13,14,15], fibromyalgia [16,17], athletes engaging in extreme and high-risk sports [18], students of the arts [19], obese individuals and people with eating disorders [20], postpartum depression [21], and severe neurological diseases [22,23]. However, with respect to a diagnosis of bipolar disorder (BD) conducted according to the criteria of the international classifications in use at the time, a series of studies clarified that the screeners, specifically the MDQ, produced false positives [24]. In fact, individuals scoring positive on the MDQ did not frequently receive a diagnosis of bipolar disorder in clinical settings but, rather, of post-traumatic stress disorder, borderline personality disorders, specific phobias, attention deficit disorder, alcohol and drug use disorders, impulse control disorder, and eating disorders [24,25,26,27]. Therefore, the episodes of energy boost identified by BD screeners (i.e., answering positively to a series of short questions on the MDQ and other screeners) did not coincide with episodes of hypomania. However, although the screener was not accurate compared to the golden standard, it identified an area of “positives” of clinical interest. For this reason, it was observed that even if the individuals scoring positive on the MDQ did not fulfill all the criteria for a diagnosis of BD, they presented some homogeneity with BD [4], not only regarding sex and age distributions but also functioning, with a high level of distress, low social functioning, and a lower perception of quality of life [28,29,30].

It was also found that MDQ positives, consistent with people with BD, showed an elevated risk of suicide, particularly young people who were likely to develop a diagnosis of BD in the future [31,32]. The diagnoses (different from those of bipolar disorder) affecting MDQ positives were conditions well known to be frequently associated with BD [4] and notoriously clinically identified about ten years before the onset of the co-occurring BD [33]. There is a vast literature that focuses on co-morbidity with BD and, often, on the temporal sequence (according to the late recognition of diagnoses of BD) with the mental health disorders indicated by the research on the inefficacy of the screeners, specifically post-traumatic stress disorder [34,35,36], borderline personality disorders [37,38,39], specific phobia [40,41,42], attention deficit disorder [43,44,45], alcohol use disorders [46,47,48], substance use disorders [49,50,51], eating disorders [52,53,54], and impulse control disorder [55,56,57].

At that point, the opinions of the experts seemed divided. Most clinicians, even after the evidence of the low accuracy of the screeners and, therefore, of the low validity of the population studies suggesting the expansion of the area of BD, decided that the diagnosis of bipolar disorder should be related to a set of “severe” conditions” and separated from the depressive ones [58]. In contrast, according to a minority of “neo-Kraepelinian” experts, the focal issue consisted of starting to verify if the cases of bipolar disorder identified in clinical settings were the emerging tip of an iceberg with a significant amount of “submerged conditions” classified as depressive but with subthreshold features and treatment response profiles of BD, thus an area of the so-called “bipolar spectrum” [59,60,61,62].

However, new evidence could suggest the need to verify a new heuristic hypothesis. The results of a recent study confirm the poor validity of the MDQ as a screener since its scores do not correlate with the presence of genetic variants associated with bipolar disorder [63]. This evidence was challenged by new findings, which showed that some genetic characteristics associated with bipolar disorder were found to be frequent in older adults without bipolar disorder but with adaptive traits of hyperactivity and novelty seeking [64,65,66]. The frequency of the same gene in hyperactive people without BD does not contradict an association between BD and that genetic variant, but it makes a linear correlation between the MDQ score and genetic risk indices unlikely. This hypothesis was verified by studying the accuracy of screeners on both the MDQ score and, in parallel, the presence of a genetic variant known to be associated with bipolar disorder, keeping the clinical diagnosis of bipolar disorder as the Gold Standard [65]. Neither of the evaluated tools (the MDQ or the genetic test) had sufficient accuracy to be used as a screener, although both had some validity [67]. Interestingly, the level of concordance between the two screeners was shallow; in fact, each screener seems to have identified a component factor that was not associated with, or was even independent of, the other one [65]. The work on the absence of correlation between the MDQ score and the genetic risk [63] presented, however, another relevant element, i.e., the MDQ positivity, which was found to have a high genetic correlation with other conditions, such as post-traumatic stress disorder, anxiety disorders, and, specifically, insomnia, one of the most relevant elements of the so-called “social rhythm”, whose psychopathological relevance has been recently emphasized [68,69].

In essence, if the increase in energy (detected by the MDQ) does not reach the threshold to be identified with an episode of hypomania on a clinical level and does not give rise to depressive “rebounds,” it is closely associated with post-traumatic and anxiety disorders and is even positively correlated with the genetic variants associated with these disorders. This association is not found in bipolar disorder. Even more closely, MDQ positivity was associated with the presence of altered social rhythms.

Another interesting theme concerns the relationship between a positive score on the MDQ and an impaired perception of Health-related Quality of Life. A community survey found that the impairment in health-related quality of life in individuals who screened positive for Bipolar Disorder on the MDQ was largely only related to scoring positive on the screener, regardless of comorbid conditions and even when a diagnosis of Bipolar Disorder based on a semi-structured psychiatric interview was excluded [30]. Further evidence also confirmed that a positive score on the screener identifies an area of people with no pathologic mood status but with a low level of quality of life [29]. It has been underlined that QoL is more impaired in bipolar disorder than in other mood disorders and anxiety disorders. It was also found that, in people with bipolar disorder, QoL was negatively associated with depressive symptoms and episodes. On the other hand, QoL appears to be a meaningful and important indicator of outcome and recovery in Bipolar Disorder. For this reason, it was initially believed that the impairment of QoL in individuals with positive scores was an indication (if not proof) of the so-called spectrum of bipolar disorders, even in the absence of a diagnosis of bipolar disorder.

Thus, some questions arise:

(1) Can the impairment be found to be associated with a positive score on the MDQ and, specifically, the impairment of quality of life, which is evidently not attributable to the presence of a bipolar disorder (given the high frequency of “false positives”), be attributed to the concomitance of associated disorders, or is it an “intrinsic” characteristic of people scoring positive on the MDQ?
(2) If the impairment was not specifically associated with positivity with other disorders, would it be possible to think that a positive score on the MDQ (and the associated rhythm dysregulation) could be a stress factor constituting a trigger for many disorders and, therefore, a co-factor that triggers the eventual genetic disposition?

To test the hypothesis of an “intrinsic” property of the MDQ to identify people with a bad quality of life regardless of any associated disorders, we worked on the database of a previous epidemiological Italian nationwide survey, which measured the frequency of positive scores on the MDQ in the community as well as, in all the interviewees, the presence of mental health disorders and the individual impairment/well-being as a measure of the quality of life [9].

Our hypothesis is that people with a positive score on the MDQ and without the co-presence of mood disorders show a worse level of health-related quality of life (HRQoL) compared to a control sample of the same age and sex and with psychiatric comorbidity.

## 2. Materials and Methods

### 2.1. Design

A case-control study built on a database of community surveys.

### 2.2. Recruitment Methods and Study Sample

The database from which the study sample was extracted was randomly drawn after stratification by age and sex from selected urban and rural municipality records (adult population) in six different Italian regions (3 from the South, 1 from the Center, and two from the North of Italy). A sample of 4999 people was drawn. A more detailed description of the community survey has already been published [9]. From this database, we extracted all the people scoring positive on the MDQ (cases), and we excluded (exclusion criteria for cases) all the individuals who had co-morbidities with a diagnosis of mood disorder. We then searched, for each MDQ-positive person, for all MDQ-negative people of the same age and gender who had the same co-morbidity for non-mood DSM-IV psychiatric disorders (eligible controls). The exclusion criteria for controls were an MDQ borderline score of 6 or 7. For each MDQ-positive case, a cell with all the people eligible to be matched as a control for that case was created. Then we randomly selected a control for each case. We excluded from the analysis all the MDQ-positive people who had co-morbidities that did not allow for a match.

### 2.3. Study Tools

Basic demographic data were assessed on an ad hoc basis.

The presence of DSM-IV psychiatric disorders was confirmed by means of the Advanced Neuropsychiatric Tools and Assessment Schedule (ANTAS), a validated semi-structured clinical interview [70].

The Italian version of the Mood Disorder Questionnaire (MDQ) was used to assess lifetime hypomanic episodes or episodes of augmentation of energy [7].

The perceived HRQoL was assessed by means of the Health Survey Short Form (SF-12). The SF-12 explores dimensions such as physical functioning, emotional state, pain, general health, vitality, social activity, and mental health [71].

### 2.4. Ethics

The protocol for the Italian community survey was approved by the ethical committee of the Italian National Health Institute (Rome). The approved protocol envisaged the possibility of conducting case-control studies on the database to investigate specific working hypotheses. Informed consent was signed by each candidate.

### 2.5. Statistical Analysis

The comparison between means and standard deviations of the SF-12 scores between cases (MDQ+) and matched controls (MDQ−) was conducted by means of statistical parametric analyses (ANOVA). The comparison between the number of people with low levels of SF-12 cases and controls was conducted by means of the Chi-squared test.

## 3. Results

Applying the methodology described above, forty-one pairs of positive cases (MDQ+) and controls (MDQ) matched for sex (twenty-three males), age (42.95 ± 14.34), and psychiatric co-morbidity (eighteen without a psychiatric diagnosis, one with specific phobia, one with anorexia, and one with obsessive-compulsive disorder) were selected (see Table 1). The scores on the instrument that measured the health-related quality of life (SF-12) were clearly lower in cases compared to controls, F(1,81DF) = 31.492 *p* < 0.0001, with an average difference of about six points (see Table 1).

The differences remained largely unchanged for people aged 50 and over. A drastically low score (35 or less) on the SF-12 was markedly higher among cases compared to controls (19/41 or 46.3% versus 3/30 or 7.9%, OR = 10.93 (2.90–41.19). The difference remained significant in people aged 50 or more, but, in this case, the OR decreased fairly (9/17 = 52.94% against 3/17 = 17.6%, OR = 5.25 (1.09–25.21) (see Table 2).

## 4. Discussion

The study shows that people who live in the community and have a positive score on the MDQ screener, even in the absence of a diagnosis of mood disorders, show significantly lower scores on the health-related quality of life questionnaire SF-12 compared to people of the same age and of the same sex with an equal diagnosis of psychiatric disorders not related to mood disorders. These differences remain high in the sub-sample of people over the age of 50, in whom the onset of a mood disorder is more unlikely. The frequency of extremely low SF-12 scores (less than or equal to 35) is markedly higher in cases compared to controls. This difference remains statistically significant for people aged 50 or older.

In the debate whether a positive score on the MDQ selects an area of “malaise” attributable to a series of disorders often present in people with positive scores on the MDQ who do not have BD disorders, (post-traumatic stress disorder, borderline personality disorders, specific phobia, attention deficit disorder, alcohol, substance use disorders, eating disorders, and impulse control disorders) or if a positive score on the MDQ may be considered a “subthreshold” form of bipolar disorder in people who may later develop bipolar disorder, then a third hypothesis can be advanced, i.e., that a positive score on the MDQ does not identify a generic area of malaise linked to “other” disorders, but a specific syndrome characterized by a considerable amount of suffering and not attributable to other disorders, which might represent a trigger for the previously mentioned disorders with which a positive score on the MDQ is associated, probably including, in the most serious forms and in people with genetic risk, bipolar disorder.

It must be kept in mind that a difference of six points on the total score of the health-related quality of life scale, i.e., in relation to dimensions such as physical functioning, emotional state, pain, general health, vitality, social activity, and, specifically, mental health [72], is a notable difference, even greater than that highlighted between those people suffering from certain psychiatric and non-psychiatric disorders and people that do not have these diagnoses. Comparing studies taken from the same database, the “burden” of MDQ positivity, understood as a difference in the health-related quality of life against controls without MDQ positivity, is lower than that highlighted with a similar methodology in disorders such as multiple sclerosis [73], fibromyalgia [74], and atherosclerosis [75], but is even higher than the “burden” of pathologies such as specific phobia [76] and panic disorder [77], and is comparable to the burden of major depression [78] or eating disorders [79]. It is therefore a condition that is associated with a considerable impairment not attributable to other associated psychiatric diagnoses. However, what other features could be hypothesized to be associated with this MDQ positivity in addition to the lifetime episodes of increased energy identified by the instrument? Importantly, other studies have found that a positive score on the MDQ is closely associated with sleep disturbances, regardless of the presence of bipolar disorder [53]. It is also well known that sleep dysregulation and energy dysregulation, as identified by the MDQ, are often associated with other social rhythms, such as the rhythms of eating and social relationships [79,80]. In support of this hypothesis of the consistency of a “Dysregulation of Mood, Energy, and Social Rhythms Syndrome” (DYMERS), some evidence was accumulated in the COVID era. The emergency induced by the pandemic and, above all, the consequent lockdown have, in fact, shown, on a clinical level, how the dysregulation of social rhythms such as energy, sleep, food rhythm, and social rhythms could influence negatively (or positively, in the case of maintaining good rhythms) not only mood disorders [69], but also work-related stress [81] and general well-being in people without previous disorders [82,83].

The heuristic hypothesis that produces the reading of these data is that three levels of energy increase can be identified:

(1) Episodic increase in adaptive energy (typical, for example, of the sports stars that perform excellently even if they do not sleep the day before the race due to the hyper-activation [84]), which a previous study identified in healthy and well-adapted hyperactive elderly people who possessed one of the genetic variants also found to be associated with bipolar disorder [64,65,66].
(2) Episodic increase in energy that enters the stress area and is associated with the dysregulation of social rhythms (sleep, eating, and relational). This condition is typical of MDQ positivity and is associated with anxiety disorders and post-traumatic stress disorder (more closely than bipolar disorder), but not exclusively, as it is even found alone in MDQ positives without co-morbidity. The increase in energy is still finalized, but inefficacy in resolving issues (hence short-circuiting the stress as, for example, seen paradoxically in some cases of burnout/occupational stress syndromes [85]) may impair the health-related quality of life.
(3) Hypomania with mood dysregulation and bipolar disorder implies a strong out-of-control hyperactivation that loses relationships with adaptive goals and often rebounds into depression. In this case, the increase in energy is such that it can be clinically identified as a hypomanic episode. This condition does not only require greater stress and environmental factors but also, possibly, a specific and additional genetic risk component. In fact, it has been shown that genetic risk and a positive score on the MDQ could be independent components of BD [67]. A predisposition to hyperactivity could be the substrate of the disorder if activated by great stress.

From this point of view, the neo-Kraepelinian concept that sees the primacy of mania and considers depression to be like the ashes after the fire of mania in bipolar disorders is not totally in contradiction with this hypothesis [60].

Indeed, if it can be hypothesized that a serious fire can cause the ash (i.e., severe and chronic depression) and that a fire can re-explode from the ash as in a volcano after a really important stimulus (a switch from drugs or considerable stress), it can also be conceivable that, in predisposed individuals, a less violent fire can cause consequences such as rhythm dysregulation and vulnerability to stress, even without necessarily depressive symptoms.

The limits of this study lie in the fact that the aim was to preliminarily verify a heuristic hypothesis on a historical sample, whose aim was to accumulate preliminary data for supporting specific ad hoc research on the actual consistency of the “Dysregulation of Mood, Energy, and Social Rhythms Syndrome” (DYMERS). The methodology of exclusion of people with multiple co-morbidities due to the absence of matched controls may have generated a bias because the cases with greater impairment were excluded from the analysis, but, at the same time, it allowed for a perfect balance between cases and controls matching all potential psychiatric diseases influencing the health-related quality of life. Having excluded the most serious cases with the presence of a positive score on the MDQ also goes against the working hypothesis, according to which a positive score on the MDQ is associated with a serious impairment since a greater impact could be assumed in people who are sick for other reasons. The value of the results was, therefore, not affected.

Although QoL can also be impaired by other confounders, such as education, employment status, and marital status, only age and sex were taken into account as matching variables, as it is difficult to hypothesize that, on the basis of literature data, the influence of these confounders—which always interact with age and sex—can be crucial in determining the large difference that emerged from our analysis [86,87].

A case-control study built on a historical database did not obviously make it possible to conduct specific analyses to identify the direction of the association between the MDQ and the HRQoL scores. In fact, it would be theoretically possible that people with a specific personality trait that manifests itself in low HRQoL are predisposed to episodes of stress and rhythm dysregulation. This limited research cannot obviously answer this question, which would require the conduct of longitudinal studies. However, this aspect should be correctly reported. The study, given the importance of the health burden of MDQ positivity independent of other psychiatric diagnoses, suggests an urgent need for ad hoc research.

## 5. Conclusions

In the debate whether a positive score on the MDQ selects an area of “malaise” due to the presence of disorders differing from Bipolar Disorders, or if a positive score on the MDQ may be considered a “subthreshold” form of bipolar disorder in people who may later develop bipolar disorder, a third hypothesis can be advanced, i.e., that a positive score on the MDQ identifies a specific “Dysregulation of Mood, Energy, and Social Rhythms Syndrome” (DYMERS), characterized by a considerable amount of suffering and not attributable to other disorders, and which might represent a trigger for the previously mentioned disorders with which a positive score on the MDQ is associated, probably including, in severe conditions, bipolar disorder.

## Figures and Tables

**Table 1:** Comparing SF-12 scores in cases and controls.

	Age	Sex	MDQ+ Score	MDQ− Score	Case-SF12 Score	Control- SF12 Score	Diagnosis
1	21	M	9	0	45	45	N (None)
2	22	F	10	3	39	40	N
3	23	M	9	3	43	42	N
4	24	F	8	0	40	40	Specific Phobia
5	25	M	8	0	35	41	N
6	25	F	11	0	34	46	N
7	26	F	8	0	38	42	Anorexia
8	27	M	8	1	27	44	N
9	27	M	8	0	40	42	N
10	28	F	11	1	24	42	N
11	29	F	9	3	27	36	Obsessive-compulsive disorder (OCD)
12	30	M	8	5	45	43	N
13	31	M	13	0	23	43	N
14	32	F	8	1	39	38	N
15	33	M	8	0	37	44	N
16	35	M	14	0	38	45	N
17	36	M	13	1	34	38	N
18	38	F	10	0	43	44	N
19	41	F	12	0	35	41	N
20	42	F	10	0	40	42	N
21	43	M	10	5	29	43	N
22	45	M	11	0	29	44	N
23	49	M	8	1	42	46	N
24	49	F	10	0	33	42	N
25	50	M	10	0	25	46	N
26	50	M	8	0	47	44	N
27	50	F	11	1	39	34	N
28	52	F	8	0	36	44	N
29	54	M	8	0	42	41	N
30	54	F	8	1	36	44	N
31	54	F	9	4	31	32	N
32	57	F	13	5	40	43	N
33	59	M	11	0	23	33	N
34	59	F	8	0	35	41	N
35	60	M	9	0	33	39	N
36	61	M	10	0	33	39	N
37	62	M	8	0	31	38	N
38	63	M	8	1	32	46	N
39	63	F	9	0	29	42	N
40	65	M	8	0	43	43	N
41 (23M)	67	M	9	1	30	39	N
	42.95 ± 14.34		9.48 ± 1.68	0.90 ± 1.51	35.21 ± 6.30	41.48 ± 3.39	F(1,81DF) = **31.492** ***p*** < **0.0001**
≥50 N = 17	57.64 ± 5.34				34.68 ± 6.26	40.47 ± 4.17	F(1,33)= **15.511** ***p*** < **0.0001**
<50 N = 24	32.54 ± 8.37				35.79 ± 6.33	42.20 ± 2.46	F(1,47) = **21.828** ***p*** < **0.0001**

**Table 2:** Frequency of people with low SF-12 scores by age.

	MDQ+	MDQ−	Chi-Square	*p*	OR (CI 95%)
People with SF-12 score ≤ 35	19	3	15.903	<0.0001	10.93 (2.90–41.19)
People with SF-12 score > 35	22	38			
People with SF-12 score ≤ 35 (Only ≥50)	9	3	4.636	0.031	5.25 (1.09–25.21)
People with SF-12 score > 35 (Only ≥50)	8	14			
People with SF-12 score ≤ 35 (Only <50)	10	0	11.007	<0.0001	Inf (NC)
People with SF-12 score > 35 (Only <50)	14	24			

## Data Availability

The approved protocol envisaged the possibility of conducting case-control studies on the database to investigate specific working hypotheses.
